# The Acute Impact of the Early Stages of COVID-19 Pandemic in People with Pre-Existing Psychiatric Disorders: A Systematic Review

**DOI:** 10.3390/ijerph19095140

**Published:** 2022-04-23

**Authors:** Sandra Carvalho, Catarina G. Coelho, Bruno Kluwe-Schiavon, Juliana Magalhães, Jorge Leite

**Affiliations:** 1 Translational Neuropsychology Laboratory, William James Center for Research, Department of Education and Psychology, University of Aveiro, 3810-193 Aveiro, Portugal; 2 Psychological Neuroscience Laboratory, CIPsi, School of Psychology, University of Minho, 4710-057 Braga, Portugal; catarina-d@hotmail.com (C.G.C.); bschiavon@psi.uminho.pt (B.K.-S.); julianamsm@gmail.com (J.M.); 3 Portucalense Institute for Human Development (INPP), Portucalense University, 4200-072 Porto, Portugal; jorgel@upt.pt

**Keywords:** mental health, psychiatric disorders, COVID-19 pandemic, systematic review

## Abstract

People with pre-pandemic health conditions are more vulnerable and more likely to suffer greater psychosocial impact due to the current COVID-19 pandemic and the lockdown measures. Thus, the objective of this work was to systematically review the impact of the early stages COVID-19 pandemic on people with pre-existing psychiatric disorders. The search was performed between 23 January and 2 September 2021 in PubMed, PsycINFO, and EMBASE. A total of 4167 published results were identified; however, only 49 were included in this review. Results show that there was considerable heterogeneity among studies, which resulted in a low consensus. However, it seems that the impact of the first stage of the COVID-19 pandemic on psychiatric disorders was two-fold: (1) an overall effect, in which people suffering from psychiatric disorders in general experienced more psychological distress and anxiety when compared to people who had no psychiatric diagnosis, and (2) a condition-specific effect, namely in people suffering from eating disorders and obsessive compulsive disorders. Moreover, the current work highlights that there were also some external factors that were related to worsening symptoms. For instance, unemployment or experiencing work and financial difficulties can be a trigger for greater distress during the pandemic for people with mood disorders, and being alone and in social isolation during the COVID-19 pandemic may actually increase substance use and relapse rates. Further studies are needed to prospectively investigate the long-term effects of the current COVID-19 pandemic on people with (pre)-existing psychiatric conditions and on the onset or deterioration of psychiatric-related symptoms in a larger number of participants, as well as exploring the long-term effects of the current pandemic on mental health.

## 1. Introduction

The COVID-19 pandemic, declared on 11 March 2020 by the World Health Organization, started in China and rapidly spread across the world, producing a tremendous impact on people’s lives, especially vulnerable populations. Thus, in an effort to contain the disease transmission, governments all over the world imposed social isolation and lockdown measures, which became increasingly stricter as the number of new infections escalated with healthcare systems under increasing strain. While such coping strategies helped limit the transmission rate of COVID-19, they also dramatically impacted the world’s social and economic reality (see [1] for Africa, [2] for Asia, [3] for Europe, and [4] for the US). This gave rise to a worldwide crisis, which may have fostered far-reaching mental health repercussions on people [5,6] due to the high level of uncertainty surrounding not only their physical health, but also their social, family, professional, and financial lives [7]. Evidence from previous research on the effects of the current pandemic on mental health suggests a negative impact on mental health in the general [8,9,10,11], as well as in vulnerable populations, which is manifested in symptoms of fear, psychological distress, anxiety, and depression [12,13,14,15,16]. Despite the fact that people with pre-existing mental disorders are more at risk of developing several conditions, including a higher risk of infections, hospitalization, or death from COVID-19 [17], little is known about the impact of the current pandemic on people suffering from pre-existing psychiatric disorders.

High levels of fear, boredom, frustration, social isolation, and loneliness are stressors that play a crucial role in the exacerbation of pre-existing symptoms and/or development of new symptomatology in people suffering from psychiatric disorders [5]. For instance, several studies reported changes in eating disorders, anxiety disorders, and alcohol and substances use during COVID-19 [18,19,20,21]. Particularly during lockdowns, vulnerable populations, such as the ones suffering from pre-existing psychiatric disorders, were in a troublesome situation, as mental healthcare systems around the world had to adjust their services and limit the face-to-face evaluations and interventions, moving to telemental health support. Telemental health support defines a set of psychological and mental health services that are provided via telecommunication technologies, such as evaluations and therapies delivered through videoconferencing, telephone calls, mental health apps, and internet-delivered programs. These programs can be provided in a synchronous (i.e., real-time conferences, telephone calls) and asynchronous (i.e., not in real time—emails, online surveys, apps, etc.) manner. Despite recent evidence suggesting that telemental health support is feasible and possibly as effective as in-person services [22,23] in monitoring mental health with periodic evaluations and providing treatments, not every person had access to these services, and not all mental healthcare providers were able to adapt to these types of services. In fact, lockdown measures around the world have negatively affected the availability of mental health services to people with psychiatric disorders, with other types of health-related services being granted top priority [24]. So, with a vulnerable population at risk of increased mental and general-health-related issues, with decreased support, it is important to assess the impact of the COVID-19 pandemic on the psychiatric well-being of people suffering from pre-existing mental disorders. Therefore, the aim of this systematic review was two-fold: (1) first, to explore whether the COVID-19 pandemic is related to changes in psychiatric symptoms of people with pre-existing psychiatric disorders; and (2) second, to evaluate the impact of the COVID-19 pandemic on people with pre-existing psychiatric disorders, for instance, the effectiveness of treatment response provided to these patients during the pandemic, changes in their daily life, overall quality of life, and awareness of the current pandemic attendance to precaution measures.

## 2. Methods

### 2.1. Protocol Search Strategy

We followed the Preferred Reporting Items for Systematic Reviews and Meta-analysis (PRISMA) guidelines [25] for reporting this systematic review.

### 2.2. Search Strategy

The comprehensive literature search was performed between 23 January and 2 September 2021, using the following databases: PubMed/MEDLINE, PsycINFO, and EMBASE per the PRISMA Guidelines for reporting systematic reviews. We searched for prospective and cross-sectional studies that evaluated the impact of the current COVID-19 pandemic on psychiatric symptoms in people with pre-existing psychiatric disorders. After consulting the Mesh terms (please see supplemental material for details), our first search included the combination of three key blocks of terms related to: (1) psychiatric disorders, and (2) COVID-19 pandemic, and (3) prospective and cross-sectional studies. 

The search terms were required to be presented in the title/abstract or topic, and a filter to select only studies with humans was used. Previous literature was hand-searched to identify additional studies that were not detected during our search and that met our inclusion criteria. 

### 2.3. Eligibility Criteria

We included prospective and cross-sectional studies that evaluated the impact of the current COVID-19 pandemic on psychiatric symptoms in people with pre-existing psychiatric disorders and/or the effectiveness of treatment response provided to these patients during the pandemic, changes in daily life, overall quality of life, and awareness of the current pandemic attendance to precaution measures.

The literature was reviewed in two steps. During screening phase, all studies were imported to Rayyan (https://rayyan.qcri.org (accessed on 23 January and 2 September 2021)), a free web application for managing systematic reviews. After eliminating duplicates, three investigators (BS, CC, and JM) independently reviewed all abstracts and extracted the potentially eligible full texts of articles to Rayyan for consideration in a second inspection. The second inspection was performed by four investigators (SC, BS, CC, and JM), under the supervision of SC in order to reach consensus. Included articles were selected according to predefined inclusion criteria, and disagreements between the authors were resolved by consensus. 

The inclusion criteria were: (a) study design: prospective and cross-sectional studies; (b) population of interest: adult people with psychiatric disorders diagnosed prior to the COVID-19 pandemic; (c) measure of psychiatric symptoms: studies were included if quantitative measures of psychiatric symptoms were measured before and during the COVID-19 pandemic, with both self-reported or observed measures (e.g., telephone/online surveys with in–outpatients from psychiatric services, etc.) performed by a mental health professional; and (d) language: English-language studies. Importantly, genetic syndromes and neuropsychiatric conditions (such as dementia and cognitive impairments) were not considered as psychiatric samples. 

The remaining full texts were independently screened for eligibility by all authors, and the following exclusion criteria were applied: (a) not having reported psychiatric symptoms as an outcome; (b) not having directly accessed the target population, i.e., people with pre-existing psychiatric disorders; and (c) studies based on service data and reports and qualitative data.

## 3. Results

### 3.1. Study Selection

**Figure 1:**
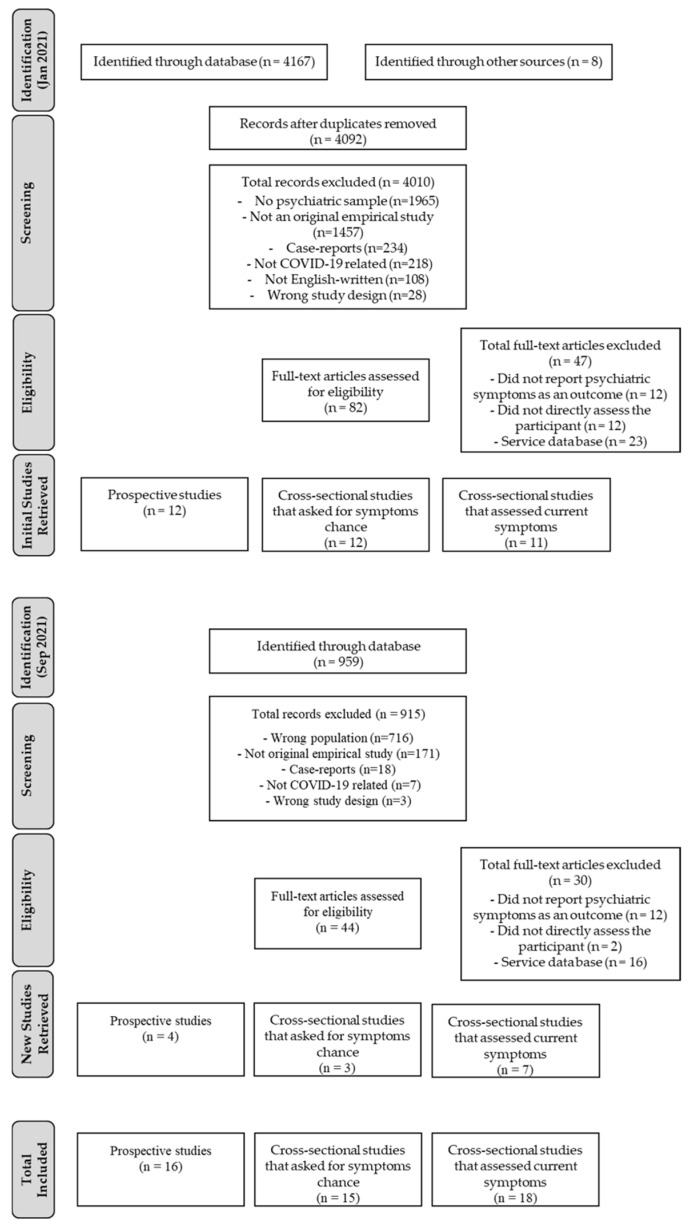
PRISMA flow diagram for the systematic review detailing the database searches, the number of abstracts screened, and the full texts retrieved for prospective and cross-sectional studies.

In the first search, a total of 4167 articles were identified (4159 articles identified through database searching and 8 through other sources). After excluding duplicates, the search identified 4092 unique entries that were screened following our inclusion criteria (see Figure 1, PRISM-based flow diagram scheme of review). In the screening phase, 4010 studies did not match the inclusion criteria and, therefore, only 82 studies were fully checked for eligibility. After applying the exclusion criteria in the eligibility phase, 35 studies remained. Concerning the updated search that was performed in September, an additional 14 studies were included after screening and an eligibility check. Therefore, a total of 49 studies were considered in this review. 

Results are organized according to the primary diagnosis: mood disorders, anxiety disorders, obsessive compulsive disorder, eating disorders, substance use and addictive disorders, attention-deficit hyperactivity disorder, schizophrenia spectrum disorders, Tourette syndrome or chronic tic disorders, insomnia disorder, somatic symptom disorders, and global impact on multiple psychiatric disorders, for those studies that studied the impact of the pandemic on multiple psychiatric conditions.

### 3.2. General Studies Characteristics

The characteristics of included studies, such as type of study design and sample characteristics, are presented in Table 1. Six (12.24%) of the studies assessed mood disorders. Two (4.08%) studies assessed anxiety disorders. Nine (18.37%) studies assessed participants with obsessive compulsive disorder. Six (12.24%) studies assessed participants with eating disorders. Six (12.24%) studies assessed participants suffering from substance-related disorders. One study assessed the effects in attention-deficit hyperactivity disorder (2.04%), and another (2.04%) study assessed participants diagnosed with schizophrenia or other psychotic disorders. Tourette syndrome or chronic tic disorder (2.04%), insomnia disorder (2.04%), and somatic symptom disorder (2.04%) were assessed by only one study each. Finally, fifteen (30.61%) studies assessed the global impact, without investigating a specific psychiatric disorder. 

The data collection methodology (i.e., telephone survey, online survey, or face-to-face survey), the country where the study was conducted, and the timeframe of the assessments are all summarized in Table 2. Most of the data collection was conducted online (42.9%); however, in eleven studies (22.4%) data were collected in a face-to-face manner, four studies (8.2%) collected the data by telephone, and thirteen studies (26.5%) used mixed approaches. The majority of the included studies were conducted in Italy (26.5%), followed by five studies (10.2%) in Germany, Spain, and China, four studies (8.2%) in the United States, three studies (6.1%) in the Netherlands, two in India and Japan (4.1%), and one in more than one country in Europe, in Europe/North America, Iran, Ireland, Israel, Taiwan, Turkey, and the United Kingdom (2%).

#### 3.2.1. Mood Disorders

Three prospective studies assessed people with mood disorders (MD) [26,29,31]. In the study by Yocum et al., a longitudinal cohort of people with bipolar disorder was compared to a group of healthy controls [26]. In the study by Pinkham et al., a mixed sample of outpatients diagnosed with bipolar disorder (I or II) with or without psychotic features or major depression with psychotic features were compared to a sample of people diagnosed with schizophrenia or schizoaffective disorder [31].

Despite the differences, both Pinkham et al. and Yocum et al. reported that there was no substantial change regarding affective and psychotic symptoms’ severity between the baseline assessment and follow-up, independently if the baseline was measured before or after the beginning of the pandemic [26]. Nevertheless, Yocum et al. showed that people with bipolar disorder reported a greater impact from the stay-at-home orders with disruptions in routines (income/employment, social support, and pandemic-related stress). Interestingly, neither the participants with bipolar disorder nor the healthy controls reported a significant change in quality of sleep [26]. This finding is in contrast with the study by Pinkham et al., which showed that sleep duration increased during the early months of the pandemic, as well as well-being [31]. However, the authors further discussed that the lack of affective and psychotic symptom exacerbation may suggest that people who suffer from severe psychiatric disorders did not experience a significant negative effect during the early stages of COVID-19 [31]. On the other hand, Di Nicola et al. showed that 74% of the sample perceived psychological distress and, curiously, that major depressive disorder predicted more severe psychological distress when compared to bipolar disorder. However, the authors did not report having assessed psychiatric symptoms before the pandemic period [29].

Three cross-sectional studies that assessed people diagnosed with MD were also included [27,28,30]. Franchini et al., carried out a study with the goal of describing the telephone-based mental illness surveillance of the quarantine effects in a sample of MD patients [30]. The authors found that frustration due to restrictions was the most represented stressor in people with MD. In addition, most of the concerns experienced by people with MD related to fear of infection and financial worries; nonetheless, the authors found very low rates of concern regarding inadequate information or lack of supplies. In contrast, Carmassi et al. investigated acute post-traumatic stress symptoms reported by patients with bipolar disorder [28]. Work and financial difficulties, besides anxiety symptoms, appeared to be positively associated with the development of post-traumatic stress symptoms. Counterintuitively, the authors reported that acute manic symptoms seemed to be protective. Regarding the study by Zhang et al., the authors also investigated the increased occurrence of post-traumatic stress disorder (PTSD) symptoms in adolescents with MDD and healthy controls [27]. Results indicate that early in the COVID-19 outbreak, adolescents with MDD were more likely than healthy controls to experience severe psychological stress and symptoms of PTSD [27].

In sum, the available literature suggests a mild to moderate worsening of depressive symptoms, as well as PTSD symptoms in people with pre-existing mood disorders. The changes triggered by the restrictions and concerns associated with COVID-19 infection and its implications (such as work and financial difficulties) may have influenced the increase in symptomatology. However, those with severe depression comorbid psychotic symptoms may not have experienced a significant negative effect during the early stages of the pandemic.

#### 3.2.2. Anxiety Disorders

One prospective study by Plunkett et al. evaluated the psychological impact of the COVID-19 social restrictions in a sample of 30 people with pre-existing anxiety disorder (AD), comparing those with anxiety-provoking triggers (such as social phobia, agoraphobia, or anxiety related to OCD) to those with anxiety not associated with specific triggers (such as generalized anxiety disorder, panic disorder, or comorbid anxiety and depression disorders) [33]. Results show that 40% of the participants reported a significant increase in anxiety symptoms, especially those with conditions associated with a “trigger”. However, clinician-rated data noted that only 26.7% of participants experiencing worsening symptoms and, surprisingly, 46.7% of participants had improved since their last clinical evaluation prior to the beginning of the pandemic restrictions. The greatest impact of the COVID-19 restrictions was related to reduced social functioning and quality of life. The presence of comorbid psychiatric or physical health difficulties was not associated with additional symptomatology or impairment [33].

The cross-sectional study by Rogers et al. provided evidence that anxiety sensitivity associated with physical and social concerns, in a sample of treatment-seeking people with AD, was associated with increased COVID-19-related worries, anxiety, functional impairment, and symptom severity. The results suggest that anxiety sensitivity is also an important vulnerability factor for people with pre-existing AD and may be an important treatment target [32].

In sum, the literature indicates a modest impact of the early stages of the pandemic on individuals with AD. The main stressors seem to be related to the decrease in quality of life, social and economic functioning, and specific concerns associated with COVID-19 infection.

#### 3.2.3. Obsessive Compulsive Disorders

Three studies longitudinally assessed people with obsessive compulsive disorders (OCD) [12,34,39]. In the study by Davide et al., changes in severity or OCD symptomatology were evaluated in a cohort of participants in full or partially remission states before and during quarantine (i.e., six weeks after the beginning of the lockdown) [12]. People with OCD showed a significant increase in the severity of both obsessions and compulsions, with participants in remission and with contamination symptoms prior to quarantine being significantly more associated with OCD worsening during quarantine. Moreover, the results evidence that living with a relative in the same house during quarantine was significantly associated with worsening in terms of psychiatric symptoms [12]. In the study by Alonso et al., the authors showed that even though 65.3% of the patients with OCD described worsening symptoms, only 31.4% reported a >25% increase in the Yale-Brown Obsessive Compulsive Scale (Y-BOCS) during the first phase of the pandemic and as compared to at least 1 year before the current pandemic [34]. About 50% of the OCD patients reported a fear of getting infected by SARS-CoV2 and stated it as a new obsession. However, only 10% of these patients reported this new obsession as their primary fear [34]. Compared to healthy controls from the same region, the study highlighted the notable risk of increased suicidal thoughts and/or acts and changes in perceived eating and sleeping patterns, as well as higher perceived levels of anxiety and depression. In the preliminary study by Matsunaga et al., the authors evaluated changes in OCD severity and symptomatology during the early stage of the pandemic in people with fully or partially remitted OCD and showed an acute impact on the severity of obsessive compulsive symptoms [39]. However, individuals with OCD symptoms severely affected by COVID-19 were significantly more likely to have a higher trait anxiety/depressive status, higher prevalence of generalized anxiety disorder, and contamination/washout symptoms.

Six cross-sectional studies investigated symptom changes in people with OCD during the COVID-19 pandemic/lockdown [16,35,36,37,38,40]. Nissen et al. verified that during the study, participants reported OCD, anxiety, and depressive symptoms, as well as intensified avoidance behavior [40]. Importantly, the occurrence of baseline aggressive/sexual obsessive compulsive thoughts and rituals increased the risk of experiencing worsening OCD symptoms during COVID-19, as measured by the time spent on obsessions and compulsions, the interference, distress, resistance, and experienced control [40]. These findings are in accordance with the studies by Tanir et al. and Hassoulas et al., which reported a significant increase in symptoms, frequency of contamination obsessions, and cleaning/washing compulsions during the pandemic period [16,35]. Additionally, the study by Benatti et al. showed that approximately one-third of the sample reported a significant emergence of new obsessions and compulsions along with a significant exacerbation of pre-existing symptoms; additionally, authors found an increase in suicidal ideation experiences, increased internet checking, sleep disturbances, avoidance behaviors, and work difficulties [37].

Nevertheless, Chakraborty et al. showed that only 6% of their participants had an exacerbation of symptoms after the COVID-19 pandemic [38]. According to the authors, the obsession with contamination may not be generalized; they point to family support, anxiety shared with the family associated with COVID-19, and frequent hand washing by other family members, as preventive factors for the exacerbation of compulsive symptoms [38].

In sum, the results shown in the literature refer to a negative impact of the COVID-19 pandemic on individuals with OCD, including the emergence of new obsessions/compulsions and suicidal ideation. Living with family members seems to enhance or protect against the worsening of symptoms, probably depending on the behavior adopted by the family member, in the face of the pandemic.

#### 3.2.4. Eating Disorders

Three prospective studies longitudinally assessed the impact of the COVID-19 pandemic on people with eating disorders (ED) [41,42,43], and showed significant ED symptom worsening [41,42,43] or significant interference in the recovery process [43]. Curiously, Baenas et al. [42] reported that the observed effect was higher in people with AN, while Castellini et al. showed that BN patients and those without remission seem to be the most vulnerable [43]. Additionally, people with eating disorders with a history of adverse childhood experiences were more likely to report stress symptoms related to the pandemic [43]. Predictive models also showed that environmental factors, such as fearing for the safety of loved ones, might lead to an increase in binge eating episodes [43], while Giel et al. suggested that binge eating episodes may be associated with worsening depressive symptoms during confinement. In the analysis by Baenas et al., the authors demonstrated that a poorer state during the COVID-19 lockdown and lower self-directedness increased the likelihood of deteriorating ED symptoms during the confinement [42]. Of note, worsening symptoms might be observed in both long and short periods, since the study of Baenas et al. [42] followed their participants in April 2020, while the study of Castellini et al. [43] followed their participants in January 2019.

Furthermore, three cross-sectional studies were also published in the same timeframe. In a study by Schlegl et al. [45], AN patients discharged from the inpatient unit responded to an online survey. A total of 70% of these patients reported increased symptomatology during the pandemic, namely concerns regarding their eating behaviors, shape and weight, and increased motivation to engage in physical activity, as well as feelings of isolation, sadness, and impatience. Moreover, despite the fact that most of the patients in the study successfully maintained their weight, many reported increased anxiety and depressive symptoms, with decreased quality of life [45]. In a similar study, Schlegl et al. [46] also evaluated the effects of the COVID-19 pandemic on people with BN. According to the authors, people suffering from BN experienced increases in depression and general psychopathology symptoms, as well as decreased quality of life and worsening ED symptomatology [46]. Interestingly, Fernández-Aranda et al. [44] showed that the impact of the pandemic may be distinct, based on the diagnosis. People with AN significantly decreased their ED and emotion dysregulation symptomatology; people suffering from obesity decreased their weight and reported less eating-related psychopathology; however, other types of ED patients reported that confinement increased their anxiety–depressive, as well as eating-related, symptoms [44].

In sum, empirical evidence suggests that the confinement associated with the COVID-19 pandemic negatively impacted people with ED. Dysfunctional coping strategies and reduced professional monitoring during the pandemic may have converged to increase symptomatology.

#### 3.2.5. Substance Use and Addictive Disorders

Two prospective studies longitudinally assessed the impact of the COVID-19 pandemic on people with substance use and addictive disorders (SUAD) [6,47]. One study showed that participants who had a moderate to severe cannabis-related disorder increased cannabis use during the first months of lockdown [6]. Moreover, although approximately 30% of the sample, including controls, lost their jobs, there was no evidence for a change in substance use severity and no changes in symptoms of depression, anxiety, or sleep problems, suggesting, as discussed by the authors, that the impact of the lockdown on the mental health of cannabis users might have been minimal [6]. This is consistent with the data from the study by Blithikioti et al., in which most participants did not report changes in the frequency of substance use [47]. However, for those who reported changes in substance use, there was a decrease in the consumption of all substances (tobacco, alcohol, cannabis, cocaine, methamphetamine, opioids), excluding sedatives. Furthermore, contrary to what was evidenced in the previous study, more than 50% of the participants reported an aggravation of depressive and anxious symptoms. Deterioration was associated with trauma exposure, female gender, perceived stress and isolation, income reduction, and alcohol use [47].

Four cross-sectional studies that investigated the impact of COVID-19 on individuals with SUAD were included [48,49,50,51]. According to Lev Bar-Or et al., who recruited participants among adults treated at a public outpatient addiction treatment clinic, people with SUAD reported an increase in the use of substances and addictive behaviors during the COVID-19 quarantine, including sedatives, pornography, and gambling [49]. On the other hand, around 16% of the participants reported having decreased use of at least one substance, which, according to the authors, may reflect the lack of social opportunities to access sellers and/or financial uncertainty [49]. Another study carried out by Chappuy et al., with SUAD patients, evaluated the impact of lockdown on the addictions of patients to analyze anxiety, mood, and sleep as factors related to consumption, and to examine whether the lockdown period led to a change in their condition [48]. Regarding tobacco, cannabis, anxiolytics/benzodiazepines, methadone, and buprenorphine, >50% of participants reported maintaining the same consumption level. However, a significant percentage of participants reported having increased their consumption of alcohol (29.2%), psychostimulants (36.2%), and heroin (39.6%) [48]. Moreover, a considerable part of the sample (48.7%) also reported an increase in addictive behaviors (pornography, gaming, gambling, shopping). Concerning the perception of changes in mood, 30.3% of the patients experienced worsened mood, and 42.2% felt more anxious. Regarding the quality of sleep, 35.3% slept less [48]. Yazdi et al. found that 38% of participants maintained substance consumption, and 32% relapsed during the pandemic. In addition to that, the authors reported that psychosocial COVID-19 factors (e.g., isolation, anxiety, and depression) and living alone also lead to a higher risk of relapsing; they added that they also found positive associations between alcohol consumption, craving, and PTSD symptoms [51]. These findings are in accordance with the study by Martinotti et al., which found a positive correlation between craving and depressive symptoms, anxiety, and traumatic stress [50]. Additionally, the author reported that 22.9% of the sample showed moderate to severe depressive symptoms, while 30.1% of the sample showed moderate to severe anxiety symptoms [50].

In sum, the literature suggests mixed results, ranging from improvement to worsening of symptoms. Thus, the decrease in consumption was associated with the lack of social opportunities regarding access to sellers and/or financial uncertainty, while the increase in consumption was related to psychosocial factors associated with COVID-19 (i.e., isolation, anxiety, and depression).

#### 3.2.6. Attention-Deficit Hyperactivity Disorder

Only one cross-sectional study directly assessed patients with attention-deficit hyperactivity disorder (ADHD). In this regard, Becker et al. [52] examined remote learning practices and difficulties during the initial stay-at-home orders during the pandemic, in adolescents with and without ADHD. The authors showed that adolescents with ADHD experienced significantly more difficulties with remote learning, associated with less engagement in adolescent routines, a greater negative effect, and more difficulty concentrating, than adolescents without ADHD [52].

#### 3.2.7. Schizophrenia Disorder

One prospective study investigated the influence of social isolation on the psychological characteristics of hospitalized patients with schizophrenia disorder (SD) [53]. The authors compared a group of patients that were medically isolated, which reduced their daily life activities, and of the upmost importance, reduced their interpersonal communication. In general, Ma et al. found that inpatients suffering from social isolation had higher levels of psychological stress and more severe anxiety and depression than those that were not isolated [53]. Additionally, inpatients suffering from social isolation also have higher levels of C-reactive protein (increased levels of circulating C-reactive protein is related to inflammation response) when compared to the non-isolated group.

In sum, the preliminary evidence of the psychological impact of social isolation due to pandemic-related-restrictions on hospitalized people with SD showed that those in social isolation reported increased levels of anxiety and decreased sleep quality.

#### 3.2.8. Tourette Syndrome or Chronic Tic Disorder

In a cross-sectional study by Mataix-Cols et al. [54], a total of 178 individuals with Tourette syndrome (TS) or chronic tic disorder (CTD) were evaluated regarding tic severity. Approximately half of the participants reported increased tic severity—little (33%) or much worse (16%)—since the outbreak. Increased stress and anxiety related to preoccupations about finances, the future and family, confinement and lack of physical exercise/activity, fewer distractions, change or lack of routines were also correlated to perceived tic worsening. The result of this study also highlights additional challenges to these specific patients, which may have contributed to the increased stress and tic worsening, such as the possible stigma related to coughing tics, exacerbation of the tics while using a mask, and an increase in self-injury behaviors [54].

#### 3.2.9. Insomnia Disorder

One cross-sectional study included patients with an insomnia disorder (ID). Cheng et al. (2021) intended to assess the effect of a digital cognitive behavioral therapy insomnia (dCBT-I) program and the impact of the pandemic on individuals with pre-existing ID. Results show that the dCBT-I treatment increased health resilience during the COVID-19 pandemic. However, most participants in this sample reported that the pandemic had a direct impact on their lives, namely: moderate to severe insomnia; moderate general stress, as well as clinically significant stress associated with COVID-19; and moderate depressive symptoms [55].

#### 3.2.10. Somatic Symptom Disorder

In one cross-sectional study by De Nardi et al. [56], the authors evaluated the impact of the COVID-19 lockdown on a sample of adolescents with and without somatic symptom disorder (SSD). The SSD group, during the lockdown, reported a significant reduction in physical symptoms, as well as amelioration of both depressive and anxiety symptoms, as compared to healthy controls [56].

#### 3.2.11. Global Impact on Multiple Psychiatric Disorders

Four prospective studies assessed participants with general psychiatric disorders [59,61,67,69]. In the study by Marchitelli et al., they investigated the psychological and psychosocial variables that might predict weight gain in patients affected by overweight/obesity, with and without a psychiatric diagnosis, during lockdown [67].

Thirty-one participants from each group affected by overweight/obesity reported weight gain during lockdown [67]. However, weight gain predictors were different for each group, with stress and lower severity of depressive symptoms for people without a psychiatric diagnosis and binge-eating behaviors for people with a psychiatric diagnosis. The results also have important implications for the development of tailored interventions for those affected by overweight/obesity, with or without psychiatric conditions that can be further investigated in order to provide tailored interventions targeting those at risk for night-eating syndrome [67].

A large-scale study by Pan et al. evaluated the impact of the pandemic on three cohorts of participants with pre-existing psychiatric conditions that have been followed since 2004, as compared to healthy volunteers [69].No overall increase in symptom severity was observed for those with severe pre-pandemic psychiatric disorders [69]. Interestingly enough, people with the most severe psychiatric disorders showed an average significant decrease in symptom severity. Authors concluded that pre-existing illness did not necessarily seem to predispose people to a greater level of emotional reactivity during the COVID-19 pandemic. Indeed, the authors even reported that people with the greatest pre-pandemic burden on their mental health showed a slight decrease in symptoms [69].

Mergel et al., conducted a study on multiple psychiatric disorders, including participants with chronic, acute, or no mental disorders [59]. They studied social participation, social inclusion, and psychological well-being before the beginning of the pandemic, as well as during and after strict lockdown restrictions. People suffering from chronic mental conditions did not appear to be particularly affected by either the restrictions imposed or the imposed social distancing [59]. However, these participants were already at a high level of deficit in the surveyed areas at the initial timepoint of the survey. Regarding participants’ mental health status, these groups showed high stability over time. Relative to participants with acute mental disorders, who were receiving treatment at the time of initial assessment, actually improved in the follow-up assessments, even in the presence of the restrictive measures due to the pandemic [59]. Overall, the results suggest resilience towards the social isolation measures in these groups. Interestingly enough, people without a chronic or acute mental disorder did experience some transient impairments in their daily life due to the restrictions imposed by the pandemic; however, by the time of the second follow-up, when restrictions were largely lifted, these impairments were almost absent [59].

Additionally, Tundo et al., carried out a study that included participants with MD, AD, and OCD [61]. The main objective was to examine the psychological and psychopathological impact of pandemic-related stress on patients with pre-existing disorders. Overall, the study suggests that there was a limited impact of the COVID-19 pandemic on these patients [61]. Likewise, the reaction in half of the patients was similar to the one of people without mental disorders. Only a limited number of participants (i.e., 6%) experienced worsening symptoms or the emergence of a new episode during the pandemic, while only 11% raised concerns about changes in terms of lifestyle and fear of becoming ill. Interestingly enough, one out of three patients suggested positive aspects of the quarantine. The only predictor in terms of risk of relapse/worsening symptoms was the disorder, with people suffering from OCD being more prone to this risk when compared with people with MDD [61].

Eleven cross-sectional studies that investigated the impact of COVID-19 on the general psychiatric population were included [57,58,60,62,63,64,65,66,68,70,71]. In the study by Favreau et al., the authors included participants with MD, AD, OCD, PTSD, AN, BN, and borderline personality disorder [57]. Patients had higher deterioration scores in depressive (>55%) and anxious (>40%) symptoms, and sleep quality (>40%); a total of 27.9% reported compromised treatment; compared to patients with ED and MD, patients with AD showed greater resilience to contact restrictions applied due to the pandemic; and AN and PTSD patients experienced greater utility for daily structuring than MD patients [57]. Regarding the investigation by Ting et al., about 77% of participants reported an increase in emotional problems due to COVID-19, with implications for their daily functioning. Additionally, 45.2% of participants had symptoms of PTSD [60]. Additionally, as reported by Favreau et al., participants had high levels of depressive symptomatology related to the pandemic [57]. The study by Vissink et al. [62] included patients with SPD, MD, developmental disorders, and personality disorders, among others. However, due to the sample size, the authors essentially divided the patients into two groups, the SPD and MD group. In general, patients reported decreased global and mental health, as well as the presence of depressive symptoms and loneliness, as evidenced in previous studies [57,60]. Nonetheless, the results suggest that the relapse impact and measures were most notable for patients with MD. Moreover, Imai et al. carried out a study on a general psychiatric population that sought to analyze the psychological impact of plastic masks/partitions for the prevention of infection, anxiety over infection, and doctor–patient relationships during the pandemic [58]. The results suggest that there was no change with regard to communication when the doctor was wearing a plastic mask/partition. In addition, most participants responded that anxiety about being infected was reduced or simply not changed by the doctor wearing a plastic mask/partition. Additionally, the use of plastic masks/partitions had beneficial effects in reducing anxiety for infection among patients with psychiatric disorders [58]; however, such an effect was smaller in older patients. Furthermore, participants with shorter treatment durations reported greater barriers of communication due to the use of plastic masks/partitions than those who had been in treatment for longer periods [58].

Burrai et al. [63], Hao et al. [65], and Iasevoli et al. [66] compared psychiatric patients to healthy controls, and in the three studies, the authors verified that psychiatric patients reported significantly higher anxiety and stress. Burrai et al., also found that psychiatric patients and controls differed in COVID-19 risk perception and with respect to worries about their health and virus infection, with the first group scoring higher on both scales [63]. Iasevoli et al. also found that patients with mental disorders were four times more likely to perceive high pandemic-related stress and had a 2–3 times greater risk of severe anxiety and depressive symptoms [66]. Additionally, Hao et al., verified that more than one-third of psychiatric patients might fulfill the diagnostic criteria for PTSD and that about one-quarter of psychiatric patients suffered from moderate to severe insomnia [65].

Chang et al. and Porcellana et al. conducted face-to-face interviews to investigate the potential factors explaining preventive COVID-19 infection behaviors among individuals with mental illness and to evaluate the relationship between traumatic aspects of the COVID-19 emergency and clinical correlates, respectively [64,70]. Chang et al. found that the higher the trust in COVID-19 information, the more engaged the participants were in preventive COVID-19 infection behaviors, while a greater fear of COVID-19 was associated with less COVID-19 preventive behaviors, highlighting the necessity of improving trust in COVID-19 information sources for individuals with mental illness [64]. However, beyond observing a high level of distress among patients in contact with mental health services during COVID-19 emergency periods, Porcellana et al., also found that psychotic patients showed poor awareness not only of their own disorder but also of the pandemic-related problems, which might decrease adherence to precautionary measures [70]. Additionally, the authors report an association between the general state of mental health and personality disorders. Zou et al., on the other hand, investigated the prevalence of psychiatric symptoms and pain in clinically stable older patients with and without psychiatric disorders during the COVID-19 pandemic [71]. The findings show that almost half of the participants (47.1%) with psychiatric disorders reported fatigue during the COVID-19 outbreak, which has a direct impact on quality of life and more severe symptoms and pain [71].

Finally, the study performed by Muruganandam et al. intended to determine the impact of the pandemic in patients with SD and MD and to identify its relationship with their knowledge about COVID-19 [68]. The study found that, overall, patients had little knowledge and awareness of the pandemic and, interestingly, 73% of the patients did not report any anxiety and fear of being infected by COVID-19. The authors also reported that only 5% of the patients reported more suicidal ideas during the lockdown [68].

Table 3 summarizes the included studies in the review, providing the main objective of each study, the assessments used during the pandemic and/or lockdown, and the brief summary of the main results. Please note that studies are organized according to the main psychiatric condition (i.e., main diagnosis) included in the study, and studies that reported the effects of the pandemic on more than one psychiatric condition were included on the global impact in multiple psychiatric disorders).

In sum, given these results, we verified that the impact of the pandemic on general psychiatric disorders depended on the disorder and that patients with severe mental illness might not have been as affected by the early stage of the COVID-19 pandemic. Overall, studies indicate that the factors that most contributed to the worsening of symptoms are concerns associated with COVID-19 and changes in daily life, financial problems, and social functioning.

## 4. Discussion

A total of 49 papers were included in this systematic review, providing preliminary evidence about the psychological impact and symptomatology and disorder-related effects of the early stage of the COVID-19 pandemic in individuals with pre-existing psychiatric disorders, such as: mood, anxiety, obsessive compulsive, eating disorders, substance use and addiction, attention-deficit hyperactivity, schizophrenia, Tourette syndrome, insomnia, and somatic.

Overall, most studies were carried out in Italy (26.5%), one of the countries in Europe with the highest number of confirmed cases (about 4.767.440) and deaths due to COVID-19 (about 132.074) (WHO, 2021) in the initial stages of the pandemic. Most studies were, therefore, carried out between April and May 2020 (38.8%), right in the initial months of the pandemic; only one study includes data collected between April and December 2020, and none of the studies included in this review performed the data collection in the year 2021. That said, it is important to understand that this review focuses on the initial findings during the pandemic, focusing on the studies that will be very hard to replicate and that are not able to track the cumulative effect of long-term exposure to the stressful situations related to the pandemic, which may be more preeminent in the later stages than the early stages of the pandemic. However, assessing the initial impact on people with pre-existing disorders is an important topic, as it can provide value information for early-stage interventions as well as data to compare to later stages of the pandemic.

Taken together, there is a substantial heterogeneity in terms of results concerning the impact of the early stages of the COVID-19 pandemic on people with pre-existing psychiatric disorders, with some studies reporting worsening symptoms, while others reporting no evidence of worsening symptoms. However, the general conclusion of this work highlights mild to moderate worsening of symptomatology and the emergence of new COVID-19-pandemic-related symptomatology in specific conditions (e.g., people with OCD).

In fact, the current evidence highlights that coping with a very stressful life event such as the global COVID-19 pandemic is certainly very demanding, not only for the general population, but especially vulnerable populations; such is the case of people with psychiatric disorders. Thus, several studies have already shown that anxiety-provoking experiences have the capability to exacerbate mental health problems, especially during a period in which both inpatient and outpatient psychiatric units suffered substantial modifications, and some of them have completely moved to digital or phone call support due to pandemic restrictions [72].

Thus, regarding the studies that evaluated the effect of the COVID-19 pandemic on individuals with mood disorders, in general, they suggest a mild to moderate impact on symptom exacerbation [26,27,28,29,30,31]. Restriction orders and interruptions in the routine seem to be one of the main stressors for the increase in terms of symptoms [26,30]; moreover, most of the concerns reported were related to the fear of infection and to financial concerns [30]. Such financial concerns were also related to the development of symptoms of post-traumatic stress [28]. In line with the findings highlighted in this review, other studies not included in this review because they did not meet the inclusion criteria have also indicated an increase in depressive symptomatology and development of symptoms of post-traumatic stress in people with mood disorders [73,74,75]. These studies noted that these changes are mainly associated with interruptions in routine [74], concerns about the health and well-being of loved ones and society, fear of dying from COVID-19, personal finances, and risk of unemployment or reduced employment [75]. In fact, social isolation, loneliness, feelings of uncertainty, hopelessness, and sadness are core symptoms associated with mood disorders that were triggered in the general population [76] and exacerbated in people with mood disorders during the coronavirus pandemic, who were more vulnerable to this events’ consequences. Additionally, several studies have already suggested a link between depression and stressful events and showed the role of rumination and cognitive bias in this relationship [77]. Since not all people with mood disorders experienced a significant negative impact of the pandemic, it would be relevant for future studies to control for such variables to evaluate which populations would be more at risk.

Concerning the effect of the COVID-19 pandemic on individuals with anxiety disorders, studies suggest that the impact varies according to the specific anxiety disorder, indicating that individuals with generalized anxiety disorder and panic disorder have higher levels of stress associated with COVID-19 [36]. However, Plunkett et al. suggested that despite patients’ self-report of worsening symptoms, compared to pre-COVID-19 results, only a small percentage of patients experienced worsening of symptoms [33]. This worsening seems to be mainly associated with reduced social functioning and perceived quality of life [32,33]. Additionally, Taylor and colleagues suggest that individuals with a primary anxiety disorder may be particularly at risk for the development of COVID-19 stress syndrome compared to individuals with mood disorders or without disorders [78,79]. In agreement with our results, Asmundson et al. point out that the greatest concerns seem to be related to the decrease in social functioning and perception of quality of life, associated with greater fear of danger and contamination, socioeconomic consequences, xenophobia, and PTSD symptoms [73].

Concerning the assessment of the impact of the COVID-19 pandemic on people with OCD, studies indicate mild [38,39] to moderate [12,16,34,35,37,40] OCD symptom worsening. The moderate worsening of OCD symptoms was essentially expressed by the increased frequency of obsessions with contamination and compulsions to clean and wash [12,16,35]. Additionally, alongside worsening OCD symptoms, some studies also reported increased comorbidity with other symptoms, such as changes in eating patterns, changes in sleep quality, suicidal ideation, increased depressive and anxious symptoms, and increased avoidance behaviors [34,40]. Alonso et al. also showed that individuals with OCD self-reported higher increases in symptoms, as compared to the results obtained with the application of the Y-BOCS by an experienced psychiatrist [34]. In a review carried out by Zaccari et al. [80], the authors found a slight to severe worsening in OCD symptoms, especially in obsessions with contagion and washing compulsions, as mentioned above. In addition to these findings, Cunning et al. also verified the development/worsening of other symptoms, such as depression, anxiety, sleep problems, and stress associated with COVID-19, among others [81]. Despite the fact that social distancing and social isolation are common features shared across several people with OCD in coping with stress related to social exposure, one could hypothesize that those factors could be somewhat protective, especially for outside triggers. However, the consequences of the pandemic are far from being only related to social distancing. The pandemic triggered new pandemic-related fears, such as worries about one’s job, money, and health problems (such as fear of coronavirus infection as demonstrated by [34]).

The included studies that assessed alterations in ED symptomatology and general psychopathology (anxiety and depression) in people with eating disorders during the early stages of the pandemic [41,42,43,44,45,46] provided evidence for worsening symptoms for those with anorexia nervosa [42,45], and/or bulimia nervosa [41,43,46], and a history of binge eating disorder [41]. This impact on symptomatology in people with AN, especially during lockdown, was associated with poor self-directedness and poorer coping strategies in dealing with stress related to lockdown measures [44]. Compared with people without a history of ED, people with AN and BN exhibited an increase in compensatory exercise during lockdown, and those with BN experienced exacerbation of binge eating episodes, which significantly interfered with the recovery process initiated prior to the pandemic. The main factors related to this worsening were fear for the safety of loved ones [43] and worsening of symptoms of depression and anxiety during confinement [41,45,46]. In a meta-analysis carried out by Sideli et al., the authors analyzed the changes triggered by the pandemic in participants with eating disorders and mental health symptoms before and during the pandemic [82]. In line with our findings, Sideli et al., showed that more than 50% of individuals with eating disorders had experienced increased ED symptoms during the pandemic, and some studies in this meta-analysis report that this increase occurred particularly in individuals with AN [82]. Additionally, worsening was also observed in anxiety and depressive symptoms in this population [82]. Taken together, these results indicate that people with ED may have been at higher risk of relapse during the pandemic, and especially during lockdown, and that vulnerability is not only associated with the diagnosis of ED but also concomitant variables, such as childhood history and insecure attachment, as well as poorer coping strategies.

Regarding the impact of COVID-19 on substance use disorders, studies have obtained mixed results. Some studies suggested that the majority of participants did not report an increase in substance use [6,47,51]. These findings are in line with the results evidenced by the European Center for Monitoring Drugs and Drug Dependence and data presented by Europol from the Global Drug Survey, which identified a decrease in drug use and an increase in the use of psychoactive drugs [83]. However, other studies point to an increase in substance consumption and addictive behaviors [48,49]. Mallet et al. also found discrepancies in the results obtained, suggesting that these depend on the countries in which the studies took place, and as such, some of the conclusions remain speculative [84].

In regard to changes in mood, anxiety, and sleep quality, most studies report a moderate to severe worsening of this symptomatology, possibly associated with the development of symptoms of PTSD, stress, and isolation, among others [47,48,50,51]. These results are in line with those of Munro et al., who report a detrimental impact, including an increase in both levels of anxiety and depression [85].

Finally, concerning the impact of the COVID-19 pandemic on general psychiatric disorders, some studies have indicated null or slight changes in symptoms, especially in patients with more severe mental disorders [58,59,61,67,68,69]. Some authors report that the reduced change in symptoms may be associated with a lack of knowledge and awareness of the pandemic or resilience facing the blocking restrictions and the subsequent policy of physical distancing, as well as the pandemic itself [59,61,68,70]. On the other hand, other investigations obtained different results, indicating a significant increase in depressive symptoms, anxiety, stress, and deterioration of sleep quality, among others [57,63,64,65,66]. These results appear to be associated with compromised treatment [57], decreased perception of quality of life and/or development of PTSD symptoms [60,62,71], and high perception of risk/stress related to COVID-19 [63,64,66].

Tsamakis et al. carried out a review that also indicates a discrepancy in results; however, contrary to our findings, found this discrepancy for individuals with general psychiatric disorders, suggesting that patients with pre-existing healthcare problems coped remarkably well with the pandemic [86]. The authors point out as possible explanations for these results the transmission mitigation strategies in place, namely the fact that staying at home can be used to develop a structured and fixed daily routine and build resilience [86]. However, a review by Murphy et al. suggests that the pandemic exacerbated symptoms in individuals with pre-existing mental illness, pointing out that the restrictions of the pandemic compromised normal daily routines, social rhythm and, as such, lead to increased levels of stress, depressive and anxious symptoms, insomnia, and suicidal ideation [87].

The worsening of symptoms in people with pre-existing mental illness seems to be related to several factors. As mentioned in some studies, this worsening of symptoms may also be associated with the fact that some patients are temporarily without psychological support or non-presential (online/telephone) support [43,88]. This claim is supported by several studies that showed a significant reduction in the numbers of patients seeking emergency psychiatric consultations [89,90,91]. Therefore, it is possible that the fear of contamination in emergency departments, deterioration in the accessibility of primary care services, an increase in outpatient activities involving telephone calls, videoconferencing, text messaging, etc., may explain the general deleterious impact of the COVID-19 pandemic on people suffering from pre-existing psychiatric conditions. Particularly concerning substance-related disorders, the literature suggests a decrease in addiction-related appointments in hospitals [92,93]. As Murphy et al. discussed, this might be partially a function of the general disruption to the healthcare system during the peak of the COVID-19 pandemic, or even an increase in outpatient virtual visits [87].

We also found some limitations across the reviewed studies, as some of the disorders were included only in one article, which makes it impossible to make broader comparisons among studies. Second, the majority of the studies assessed participants in the early stages of the COVID-19 pandemic, hence depicting a relatively short-term overview of the possible effect of the pandemic and lockdown policies on psychiatric symptoms. However, the data from these early studies could be useful for future emergency situations, or simply for use as a comparison point for other studies aiming at different goals. Future studies should review the impact of the pandemic at latter stages than the current one, as long-term exposure to very stressful events, associated with mass fatigue of the social distancing measures, fear of health complications (including fear of death), access to and availability of healthcare providers, and financial problems, among others, may contribute to an exacerbation of psychological/psychiatric symptomatology, which may have an increased impact on more vulnerable people , such as the ones with pre-existing psychiatric conditions.

In addition, here we only included studies that have directly assessed people suffering from psychiatric disorders. In other words, studies in which behavioral and emotional changes were rated by caregivers were not included. Moreover, only one study included in our review reported having assessed children and adolescents, which highlights the importance of assessing whether the deleterious effects of the COVID-19 pandemic are more pronounced in children and adolescents suffering from psychiatric disorders or neurodevelopmental disorders, such as autism spectrum disorders. It is also important for future studies to try to better understand the impact of several factors on the exacerbation (or not) of psychiatric symptoms. For instance, Türkoğlu et al., suggested that home confinement increased eveningness chronotype, sleep problems, and autism symptoms compared to the normal non-home confinement state [94]. Moreover, the current work focuses only on the available data regarding the impact of the early stages of the COVID-19 pandemic in people with pre-existing psychiatric disorders, and does not allow us to draw conclusions regarding the long-term effects of the current pandemic, namely the cumulative effect of the several measures that were taken, the economical and social changes that occurred due to the multiple waves, or the impact on people without pre-existing conditions.

## 5. Conclusions

This study shows important information about the real impact of the COVID-19 pandemic on individuals with pre-existing mental illnesses. Instead of a general effect, it seems that the impact of the early stages of COVID-19 on symptomatology varied according to the disorder. Although there was no absolute consensus among the studies and considerable heterogeneity, the current work highlights: (1) a significant impact of the pandemic and social isolation on people with pre-existing psychiatric disorders, particularly for those with eating disorders and obsessive compulsive disorders, although a pre-existing severe mental illness did not necessarily seem to predispose an individual to a greater level of emotional reactivity to the COVID-19 pandemic; (2) specifically, but not exclusively, for those people diagnosed with mood disorders, the current findings revealed that being unemployed or experiencing work and financial difficulties can be a trigger for greater distress; (3) regarding substance-related disorders, results suggest that being alone during quarantine (i.e., living alone) and social isolation during the COVID-19 pandemic may increase substance use and relapse rates; (4) one study showed that people suffering from Tourette syndrome reported a worsening in their symptoms during the COVID-19 pandemic, while another study showed that children and adolescents diagnosed with ADHD may report more remote learning difficulties; and (5) people suffering from psychiatric disorders in general experienced more psychological distress and anxiety when compared to people who had no psychiatric diagnosis. Thus, participants with mood, eating, and obsessive compulsive disorders seemed to show a more accentuated worsening of symptoms, influenced by several factors. It is important to highlight that this review is focused on the impact of the initial stages of the pandemic on pre-existing mental disorders and, as such, has several limitations arising from the studies that support these findings. More specifically, future studies should further assess the magnitude of the impact of the successive waves of the pandemic, distinguishing, if possible, the impact of several degrees of lockdown, isolation, or social distancing measures, among others. Future studies should also compare the impact on the population with pre-existing mental disorders with the impact of the pandemic on the general population, as future policy measures should take into account such potential variations. Finally, further studies are needed to prospectively investigate the long-term effects of the current COVID-19 pandemic on people with psychiatric conditions and on the onset or deterioration of psychiatric-related symptoms in a larger number of participants. Please see Table 4 for a brief summary of the main findings.

## Figures and Tables

**Table 1:** Study design and sample characteristics.

First Author (Year)	Study Categorization	Sample n (Mean Age (% Women))	Healthcare Information
*Mood Disorders*			
Yocum et al. (2021) [26]	Prospective	- BD: 345 (nr (nr)) - WDC: 68 ( nr (nr)) - Healthy controls: 147 (nr (nr))	- Participants admitted as outpatient or inpatient in clinical services
Zhang et al. (2021) [27]	Cross-sectional (current symptoms)	- MDD group: 90 (15 (80)) - Control group: 107 (15 (77))	- Outpatients - Psychopharmacological treatment
Carmassi et al. (2020) [28]	Cross-sectional (current symptoms)	- BD, PTSS: 17 (nr (88)) - BD, No-PTSS: 83 (nr (59))	- Online/telephone intervention - Psychopharmacological treatment
Di Nicola et al. (2020) [29]	Prospective	- No psychological distress: 29 (43 (48)); - Mild psychological distress: 35 (45 (51)); - Moderate to severe psychological distress: 48 (51 (58))	- Psychopharmacological treatment.
Franchini et al. (2020) [30]	Cross-sectional (current symptoms)	- Euthymic MD: 101 (62 (65))	- Online/telephone intervention
Pinkham et al. (2020) [31]	Prospective	- SPD: 92 (43 (54)) - MD: 56 (41 (71))	- Outpatients
*Anxiety Disorders*			
Rogers et al. (2021) [32]	Cross-sectional (current symptoms)	- AD: 99 (35 (67))	- Treatment-seeking patients
Plunkett et al. (2020) [33]	Prospective	- AD: 30 (39 (60))	- Outpatients
*Obsessive-Compulsive Disorders*			
Alonso et al. (2021) [34]	Prospective	- OCD:127 (42 (54)) - Healthy controls: 237 (41 (58))	- Outpatients
Hassoulas et al. (2021) [35]	Cross-sectional (changes)	- OCD: 254 (nr (nr))	NA
Khosravani et al. (2021) [36]	Cross-sectional (changes)	- OCD: 300 (36 (59)) - AD: 310 (36 (60))	- Treatment-seeking patients
Benatti et al. (2020) [37]	Cross-sectional (changes)	- OCD, worsening: 44 (40 (46)) - OCD, not worsening: 79 (40 (44))	- Outpatients
Chakraborty et al. (2020) [38]	Cross-sectional (changes)	- OCD: 84 (nr (76))	- Outpatients - Pharmacological treatment
Davide et al. (2020) [12]	Prospective	- OCD: 30 (43 (53))	- Psychopharmacological treatment
Matsunaga et al. (2020) [39]	Prospective	- OCD: 60 (42 (58))	- Outpatients
Nissen et al. (2020) [40]	Cross-sectional (changes)	- OCD clinical group: 65 (15 (63)) - Survey group: 37 (14 (67))	- Outpatients - Psychotherapy treatment - Pharmacological treatment
Tanir et al. (2020) [16]	Cross-sectional (changes)	- OCD: 61 (14 (44))	- Outpatients - Psychotherapy treatment (CBT) - Pharmacological treatment
*Eating Disorders*			
Giel et al. (2021) [41]	Prospective	- ED: 23 (nr (nr)) - Healthy controls: 19 (nr (nr))	- Outpatients - Psychotherapy treatment (CBT)
Baenas et al. (2020) [42]	Prospective	- ED, worse: 19 (33 (nr)) - ED, non-worse: 55 (32 (nr))	- Outpatients
Castellini et al. (2020) [43]	Prospective	- ED: 74 (32 (100)) - Healthy controls: 97 (31 (100))	- Outpatients - Psychotherapy treatment (CBT)
Fernández-Aranda et al. (2020) [44]	Cross-sectional (changes)	- AN: 55 (24 (89)) - BN: 18 (31 (94)) - OSFED: 14 (37 (86)) - Obesity: 34 (49 (77))	- Pre-pandemic: outpatients - During pandemic: Online/telephone intervention
Schlegl et al. (2020) [45]	Cross-sectional (changes)	- Adults with AN: 112 (25 (100)) - Adolescents with AN: 47 (16 (100))	- Outpatient, inpatient and online/telephone intervention
Schlegl et al. (2020) [46]	Cross-sectional (changes)	- Inpatients with BN: 55 (24 (nr))	- Pharmacological treatment - Online/telephone intervention
*Substance-related disorders*			
Blithikioti et al. (2021) [47]	Prospective	- SUD: 303 (49 (37))	- Outpatients
Chappuy et al. (2021) [48]	Cross-sectional(changes)	- SUD: 219(43 (22))	- Outpatients
Lev Bar-Or et al. (2021) [49]	Cross-sectional (changes)	- SUD: 92 (40 (38))	- Outpatients
Cousijn et al. (2020) [6]	Prospective	- Daily cannabis users: 120 (18–46 (nr)) - non-using controls: 63 (18–31 (nr))	NA
Martinotti et al. (2020) [50]	Cross-sectional (current symptoms)	- SUD inpatients: 56 (nr (nr)) - SUD outpatients: 97 (nr (nr))	- Outpatients. - Inpatients - Pharmacological treatment
Yazdi et al. (2020) [51]	Cross-sectional (current symptoms)	- Abstinent: 37 (51 (35)) - Consuming: 49 (48 (37)) - Relapsed: 41 (49 (27)	- Outpatient
*Attention Deficit Hyperactivity Disorder*			
Becker et al. (2020) [52]	Cross-sectional (current symptoms)	- ADHD: 118 (17 (35)) - Healthy controls: 120 (17 (46))	- Pharmacological treatment
*Schizophrenia or Other Psychotic Disorder*			
Ma et al. (2020) [53]	Prospective	- Isolated patients: 30 (43 (60)); - Non-isolated patients: 30 (45 (50))	- Inpatients
*Tourette Syndrome/Chronic Tic Disorder*			
Mataix-Cols et al. (2020) [54]	Cross-sectional (changes)	- TS/CTD: 178 (31 (57))	NA
*Insomnia Disorder*			
Cheng et al. (2021) [55]	Cross-sectional (current symptoms)	- dCBT-I group: 102 (45 (73)) - Control group: 106 (45 (84))	- Online psychotherapy treatment (CBT)
*Somatic Symptom Disorder*			
De Nardi et al. (2021) [56]	Cross-sectional (current symptoms)	- SSD group: 58 (15 (52)) - Control group: 57 (16 (46))	- Outpatients
*Multiple Psychiatric Disorders*			
Favreau et al. (2021) [57]	Cross-sectional (changes)	- GPP: 538 (70 (36))	- Inpatients
Imai et al. (2021) [58]	Cross-sectional (changes)	- GPP: 425 (53 (53))	- Outpatients
Mergel et al. (2021) [59]	Prospective	- CMD: 25–27 (50 (48)) - AMD: 29–30 (44 (60)) - WMD: 46–49 (41 (76))	- Outpatients - Psychiatric treatment - Psychotherapeutic treatment
Ting et al. (2021) [60]	Cross-sectional (changes)	- GPP: 193 (16–20 (7.3); 21–30 (14.5) 31–40 (21.2); 41–50 (21.2); 51–60 (23.8); 61–70 (10.4); >70 (1.6) (72))	- Outpatients
Tundo et al. (2021) [61]	Prospective (observational)	- No relapse/worsening: 365 (52 (59)) - Relapse/worsening: 21 (48 (67))	- Outpatients - Pharmacological treatment
Vissink et al. (2021) [62]	Cross-sectional (changes)	- GPP: 189 (40 (46))	- Outpatients
Burrai et al. (2020) [63]	Cross-sectional (current symptoms)	- GPP: 77 (47 (34)) - Healthy controls: 100 (46 (50))	- Inpatients
Chang et al. (2020) [64]	Cross-sectional (current symptoms)	- GPP: 414 (46 (44))	- Inpatients - Outpatients
Hao et al. (2020) [65]	Cross-sectional (current symptoms)	- Psychiatric patients: 76 (33 (37)) - Healthy controls: 109 (33 (62))	- Outpatients
Iasevoli et al. (2020) [66]	Cross-sectional (current symptoms)	- GPP: 205 (nr (nr)) - Caregivers: 51 (nr (nr)) - Controls: 205 (nr (nr))	- Outpatients
Marchitelli et al. (2020) [67]	Prospective	- Patients with psychiatric diagnosis: 47 (46 (77)) - Patients without psychiatric diagnosis: 63 (47 (67))	- Psychotherapy treatment - Pharmacological treatment
Muruganandam et al. (2020) [68]	Cross-sectional (changes)	- SMI outpatients: 132 (34 (52))	- Outpatients
Pan et al. (2020) [69]	Prospective	- With psychiatric disorder: 1181 (56 (67)) - Without psychiatric disorder: 336 (58 (55))	- Outpatients
Porcellana et al. (2020) [70]	Cross-sectional (current symptoms)	- GPP: 140 (50 (55))	- Treatment-seeking patients
Zou et al. (2020) [71]	Cross-sectional (current symptoms)	- Fatigue: 501 (62 (69)) - No fatigue: 562 (63 (66))	- Outpatients

Note: AD, anxiety disorders; ADHD, attention-deficit hyperactivity disorder; AMD, acute mental disorder; AN, anorexia nervosa; BD, bipolar disorder; BN, bulimia nervosa; CBT, cognitive behavioral therapy; CMD, chronic mental disorder; CTD, chronic tic disorder; dCBT-I, digital cognitive behavioral therapy for insomnia; ED, eating disorders; GPP, multiple psychiatric disorders; MD, mood disorders; MDD, major depressive disorder; OCD, obsessive compulsive disorders; OSFED, other specified feeding or eating disorder; PTSS, post-traumatic stress symptoms; SMI, severe mental illness; SPD, schizophrenia or other psychotic disorder; SSRIs, selective serotonin reuptake inhibitors; SSD, somatic symptom disorder; SUD, substance-related disorders; TS, Tourette syndrome; WDC, without diagnosis category; WMD, without mental disorder.

**Table 2:** Data collection procedure and assessment time.

Study ID	Country	Previous Assessments	Data Collection Method	2020											
				1	2	3	4	5	6	7	8	9	10	11	12
*Mood Disorders*															
Yocum et al. (2021) [26]	United States	None	- Online				o	o							
Zhang et al. (2021) [27]	China	2019	- Telephone/Online		o										
Carmassi et al. (2020) [28]	Italy	None	- Online				o								
Di Nicola et al. (2020) [29]	Italy	Dec 2019	- Online	o	o		o								
Franchini et al. (2020) [30]	Italy	None	- Telephone			o	o								
Pinkham et al. (2020) [31]	United States	2018	- T0: Face-to-face/T1: Telephone				o	o	o						
*Anxiety Disorders*															
Rogers et al. (2021) [32]	Spain	None	- Online					o							
Plunkett et al. (2020) [33]	Ireland	None	- Telephone/Face-to-face				o	o							
*Obsessive Compulsive Disorders*															
Alonso et al. (2021) [34]	Spain	None	- Telephone/Online				o	o							
Hassoulas et al. (2021) [35]	United Kingdom	None	- Online				o	o	o	o					
Khosravani et al. (2021) [36]	Iran	None	- Face-to-face/Telephone/Online						o	o	o				
Benatti et al. (2020) [37]	Italy	None	- Telephone/Face-to-face												
Chakraborty et al. (2020) [38]	India	None	- Telephone			o	o								
Davide et al. (2020) [12]	Italy	None	- Face-to-face	o	o		o								
Matsunaga et al. (2020) [39]	Japan	None	- Face-to-face				o	o							
Nissen et al. (2020) [40]	Denmark	None	- Online				o	o							
Tanir et al. (2020) [16]	Turkey	None	- Telephone/Online			o	o								
*Eating Disorders*															
Giel et al. (2021) [41]	Germany	2017	- Telephone/Online					o	o	o					
Baenas et al. (2020) [42]	Spain	None	- T0: Face-to-face/T1: Telephone				o								
Castellini et al. (2020) [43]	Italy	Dec 2019	- T0: Face-to-face/T1, T2: Online	o	o	o	o	o							
Fernández-Aranda et al. (2020) [44]	Spain	None	- Online						o	o					
Schlegl et al. (2020) [45]	Germany	None	- Online					o							
Schlegl et al. (2020) [46]	Germany	None	- Online					o							
*Substance-related disorders*															
Blithikioti et al. (2021) [47]	Spain	2019	- Online						o	o					
Chappuy et al. (2021) [48]	French	None	- Face-to-face					o	o						
Lev Bar-Or et al. (2021) [49]	Israel	None	- Face-to-face				o								
Cousijn et al. (2020) [6]	Netherlands	2019	- Online	o	o	o	o	o							
Martinotti et al. (2020) [50]	Italy	None	- Face-to-face/Online			o	o	o							
Yazdi et al. (2020) [51]	Austria	None	- Face-to-face				o	o	o						
*Attention-Deficit Hyperactivity Disorder*															
Becker et al. (2020) [52]	United States	None	- Online					o	o						
*Schizophrenia or Other Psychotic Disorder*															
Ma et al. (2020) [53]	China	None	- Face-to-face		o	o									
*Tourette Syndrome or Chronic Tic Disorder*															
Mataix-Cols et al. (2020) [54]	Europe/North America	None	- Online				o	o							
*Insomnia Disorder*															
Cheng et al. (2021) [55]	United States	2016, 1017	- Online				o								
*Somatic Symptom Disorder*															
De Nardi et al. (2021) [56]	Italy	None	- Online				o	o							
*Global impact on multiple psychiatric disorders*															
Favreau et al. (2021) [57]	Germany	None	- Online				o	o	o	o	o	o	o	o	o
Imai et al. (2021) [58]	Japan	None	- Face-to-face				o	o	o	o	o				
Mergel et al. (2021) [59]	Germany	2019	- Face-to-face				o	o		o					
Ting et al. (2021) [60]	China	None	- Online					o	o						
Tundo et al. (2021) [61]	Italy	None	- Face-to-face/Online			o	o	o	o						
Vissink et al. (2021) [62]	Netherlands	None	- Online/face-to-face					o	o	o	o	o			
Burrai et al. (2020) [63]	Italy	None	- Online				o	o							
Chang et al. (2020) [64]	Taiwan	None	- Face-to-face		o	o	o	o							
Hao et al. (2020) [65]	China	None	- Online		o										
Iasevoli et al. (2020) [66]	Italy	None	- Telephone				o								
Marchitelli et al. (2020) [67]	Italy	None	- Online				o	o							
Muruganandam et al. (2020) [68]	India	2019	- Telephone	o	o	o									
Pan et al. (2020) [69]	Netherlands	2006, 2008, 2010, 2012, 2014	- Online				o	o							
Porcellana et al. (2020) [70]	Italy	None	- Face-to-face				o								
Zou et al. (2020) [71]	China	None	- Face-to-face					o	o	o					

Note: T0, baseline measure; T1, first follow-up; T2, second follow-up. The vertical line indicates the official onset of the pandemic state on 11 March. The bullet points indicate the month in which the data were collected, and the dotted vertical line represents the beginning of the pandemic state on 11 March. o: is used to identify the month of the year.

**Table 3:** Studies’ aims and main findings.

First Author (Year)	Aim	Assessment Instruments during Pandemic and/or Lockdown	Main Results
*Mood Disorders*			
Yocum et al. (2021) [26]	To evaluate the impact of the COVID-19 pandemic and lockdown on individuals with BD, as compared to healthy controls.	Pandemic and lockdown: CIS; GAD-7; PHQ-9; PSQI.	- Both BD patients and heathy controls reported a negative impact of lockdown measures. - Over time, healthy controls recover more rapidly and with greater magnitude than people with BD. - Greater negative impact on mood symptoms on healthy controls.
Zhang et al. (2021) [27]	To evaluate the impact of the COVID-19 outbreak at 1 month after the start on the mental health of adolescents with or without MDD.	Pandemic: BDI-II; CRIES-13.	- Adolescents with MDD were more likely than those without MDD to experience severe psychological stress and symptoms of PTSD.
Carmassi et al. (2020) [28]	To investigate acute PTSS symptoms in patients with BD in a telepsychiatry service.	Lockdown: GAD-7; HAM-D; IES-R; YMRS.	- PTSS and moderate to severe depressive symptoms were reported by 17% of the patients, while severe anxiety was reported by 26%. - Acute manic symptoms may exert a protective function.
Di Nicola et al. (2020) [29]	To investigate the effects of lockdown on psychological distress perceived in people with MDD and BD. To assess the usefulness of serum 25(OH)D levels as a predictor of distress severity.	Lockdown: clinical interview; K10; biological data: serum 25(OH)D levels.	- A total of 74% perceived a form of psychological distress at the time of the study; 26% reported no likelihood of psychological distress; 31% displayed mild psychological distress; and 43% displayed moderate to severe psychological distress.
Franchini et al. (2020) [30]	To describe the telephone-based mental illness surveillance on euthymic MD patients to evaluate reactions to lockdown measures.	Lockdown: physical conditions; psychiatric conditions: emotional stressors and unpleasant experiences during the lockdown.	- The main concerns were frustration due to restrictions (76%); fears about infection (54%); financial concerns (46%); anxiety (45%); and low mood (41%). Moreover, about 30% of the participants reported somatization, increased alertness, and insomnia. - A total of 21% of the patients were self-medicated with sedatives, and 3% of the patients reported inadequate supplies.
*Anxiety disorders*			
Rogers et al. (2021) [32]	To examine the association between anxiety sensitivity with COVID-19 worry, functional impairment, and symptom severity in a community sample as compared to people with anxiety disorders.	Pandemic: ASI-3; COVID-19 screening. and symptoms; COVID-19 worry index; COVID-19 functional impairment; COVID-19 anxiety; severity of COVID-19 symptoms.	- Increased anxiety sensitivity was significantly associated with increased COVID-19-related worry, anxiety, functional impairment, and symptom severity.
Plunkett et al. (2020) [33]	To examine the impact of social restrictions on people with AD.	Lockdown: BAI; HAMA; CGI-I; CGI-S; GAF; Y-BOCS.	- A total of 50% of the participants described a deleterious effect of the COVID-19 pandemic on their mental health; 40% described a deleterious effect pertaining to their levels of anxiety. - Clinician-rated data revealed that 27% of the participants had worsened mental health prior to COVID-19 restrictions; 47% of the participants demonstrated an improvement in their mental health prior to COVID-19 restrictions.
*Obsessive compulsive disorders*			
Alonso et al. (2021) [34]	To investigate the impact of the COVID-19 pandemic on individuals with OCD, as compared to a community sample at an early stage of the pandemic.	Pandemic: clinical interview, Y-BOCS, VAS (depression/ anxiety levels); HDRS.	- Worsening of symptoms in 65% of the patients; 31% of OCD patients showed an increase of more than 25% in their pre-pandemic Y-BOCS scores; 45% developed an obsession related to the risk of getting infected by SARS-CoV2; however, only 10% reported it as being their main obsessive concern. - Increased suicidal ideation among the OCD cohort.
Hassoulas et al. (2021) [35]	- To investigate the impact of the COVID-19 pandemic on individuals with OCD.	Lockdown: OCI-R; SHAI; brief COVID-19 impact measure.	- Pandemic-related restrictions impacted mental well-being in 57% of the participants. Moreover, 22% reported impacts in terms of physical health, 10% reported impacts due to their social isolation, and 5% mentioned financial and occupational impacts.
Khosravani et al. (2021) [36]	- To compare COVID-19-related stress reactions among patients with AD and OCD.	Pandemic: CSS; PHQ-4; FCV-19S; C19P-S; SHAI; VOCI; XS; HCQ-54; OCI-R; OCS.	- Patients with OCD, generalized AD, and panic disorder might have a higher risk for COVID-19 stress syndrome and COVID-19 stress disorder when compared to other ADs.
Benatti et al. (2020) [37]	- To describe the impact of COVID-19 pandemics in a sample of patients with OCD.	Pandemic: clinical interview.	- More than 1/3 of the sample reported OCD worsening; 30% of the worsening group developed new obsessions and compulsions and 41% re-experienced past obsessions and compulsions; 66% of these patients experienced an increase in avoidance behaviors; 71% had significantly higher rates of pharmacological therapy adjustment; 9% reported suicidal ideation; and 52% reported sleep disturbances.
Chakraborty et al. (2020) [38]	- To assess the impact of COVID-19 on OCD patients.	Pandemic: interview: changes in symptoms; Y-BOCS.	- Most of the participants did not report any deterioration of symptoms due to the pandemic; 3 of the participants were in complete remission, and 2 were in partial remission. Only 2% of the participants had a >25% increase in the Y-BOCS total score.
Davide et al. (2020) [12]	- To evaluate changes due to the pandemic in a group of OCD patients that underwent psychiatric treatment before the pandemic period.	Lockdown: Y-BOCS; questionnaire on COVID-19 pandemic and life during the quarantine.	- Out of the 40% of patients that were remitted before the quarantine, 13% relapsed with clinically significant OCD.
Matsunaga et al. (2020) [39]	- To investigate the acute impact of the COVID-19 pandemic in fully or partially remitted OCD patients.	Pandemic: Y-BOCS.	- A total of 10% experienced increased symptom severity, while 7% had additional or renewed OCD symptoms associated with COVID-19. - Higher traits of anxiety, depressive status, higher prevalence of generalized AD, fear of contamination and washing compulsions were found in patients whose pre-COVID-19 obsessions/compulsions were associated with respiratory virus infection.
Nissen et al. (2020) [40]	- To examine how children and adolescents with OCD react to the COVID-19 crisis.	Pandemic: self-developed questionnaire on well-being (i.e., perceived changes in quality of life, psychiatric symptoms’ severity, avoidance behaviors).	- Clinical group: 45% reported a worsening of their symptoms, namely, 32% concerning anxiety; 34% concerning depressive symptoms; and 19% concerning avoidance behavior. - Survey group: 73% reported a worsening of their symptoms, namely, 54% concerning anxiety; and 43% concerning depressive symptoms. - In both groups there was a positive correlation between aggravation of anxiety and depressive symptoms and experienced worsening of OCD.
Tanir et al. (2020) [16]	- To investigate the effects of COVID-19-related home confinement on the profile, severity, and exacerbation of symptoms in OCD individuals.	Lockdown: CGI-S; CY-BOCS.	- A total of 54% reported an increase in symptom severity; there was a significant increase in the frequency of contamination obsessions and cleaning/washing compulsions during the pandemic; 34% of the patients reported no change in symptom severity; 11% reported a decrease in CY-BOCS scores; 56% relapsed with obsessive compulsive symptoms; and 31% returned to clinically significant levels of OCD during the pandemic.
*Eating Disorders*			
Giel et al. (2021) [41]	- To investigate ED symptoms, as well as general psychopathology, after the first COVID-19 lockdown in patients with a history of BED.	Lockdown: BDI-II; EDE-Q.	- Comparing the results obtained before and during COVID-19, there was a reduction of 50% in BED and a reduction of 14% in comorbid mental disorder. - No relations were found between binge eating frequency and self-reported distress, emotion regulation capacities, and sense of coherence.
Baenas et al. (2020) [42]	- To assess the level of deterioration in functioning and the factors that impact the adjustment of people with ED to the COVID-19 confinement.	Lockdown: EDI-2; SCL-90-R; TCI-R; YFAS-2.	- A total of 70% of the patients reported concerns related with the confinement; 26% reported non-adaptive reactions; 42% reported anxiety symptoms; and 30% reported depression symptoms. - A total of 26% of the patients had a worse symptom evolution during the confinement, while for 51% of the patients, the ED symptoms became less dominant.
Castellini et al. (2020) [43]	- To evaluate the impact of COVID-19 on ED patients, considering the role of pre-existing vulnerabilities.	Lockdown: BSI; CTQ-SF; ECR-R; EDE-Q; IES-R.	- 13% of the patients experienced full remission from baseline to pre-lockdown, and 25% of the patients achieved partial remission. - At post-lockdown assessment, 14% of the participants reported full remission, and 18% reported partial remission, whereas 62% still reported an ED diagnosis.
Fernández-Aranda et al. (2020) [44]	- The impact of confinement on eating symptomatology; and to explore the general acceptance of the use of telemedicine among people with ED.	Lockdown: CIES.	- AN patients reported less impact on eating symptoms, changes in eating habits and in emotion regulation. Obese patients reported decreased weight, BMI, and changes in eating habits; - Apart from AN and obese patients, other specified ED patients showed deterioration in eating symptomatology.
Schlegl et al. (2020) [45]	- To explore effects of the COVID-19 pandemic on ED symptoms and other psychological aspects in former inpatients with AN.	Pandemic: self-developed questionnaire on the overall impact of the COVID-19 pandemic on eating disorders symptoms and general well-being.	- Around 50% of the patients reported a deterioration of their quality of life; 17% indicated no worsening of it; 70% of the sample reported an increase in loneliness, inner restlessness, and sadness; 50% of the patients indicated fears of not being able to stop or control worries and worried that feelings get out of control; 50% of the patients reported that worries were related to infectng others and relapse; and 47% reported increases in family conflicts. No changes were reported in terms of friendships, romantic relationships, or workplace conflicts for 80% of the individuals.
Schlegl et al. (2020) [46]	- To investigate the impact of the current pandemic on patients with BN.	Pandemic: self-developed questionnaire on the overall impact of the COVID-19 pandemic on ED symptoms and general well-being.	- A total of 49% of the patients experienced worsening of ED symptomatology, while 62% had decreased quality-of-life scores. Moreover, binge eating increased in 47% of the patients, whereas self-induced vomiting increased in 36%.
*Substance-Related Disorders*			
Blithikioti et al. (2021) [47]	- To assess risk factors of adverse mental health outcomes during lockdown in a SUD population.	Lockdown: questions of the ASSIST Screening Test; CTQ-SF; LEC; DTS; BDI; STAI; perception about symptom change due to quarantine measures.	- Increased consumption of: alcohol (13%), cannabis (1%), cocaine (3%), methamphetamine (1%), and sedatives (9%). One participant reported an increase in opioid consumption. - More than 50% of the participants reported a deterioration in depression and anxiety symptoms, associated with trauma exposure, female gender, perceived stress and isolation, income reduction, and alcohol use.
Chappuy et al. (2021) [48]	- To measure the impact of the COVID-19 lockdown on the addictions of SUD; to examine how anxiety, mood, and sleep are related to consumption; to investigate changes in their condition due to COVID-19.	Lockdown: online questionnaire (all questions were related to the lockdown period in France).	- Increased consumption of: alcohol (29%), cannabis (28%), psychostimulants (36%), heroin (40%), anxiolytics (26%), methadone (14%), and buprenorphine (23%). There was a 49% increase in addictive behaviors. - A total of 61% of the patients remained stable regarding general psychiatric symptoms, while they worsened for 30% and improved for 9%.
Lev Bar-Or et al. (2021) [49]	- To investigate changes in addictive behaviors among individuals treated in a specialized outpatient addiction treatment clinic.	Lockdown: interview: initiated, increased, decreased, or ceased use of various specific addictive substances and behaviors.	- A total of 36% increased their addictive behavior or the use of at least one substance: 25% alcohol, 29% cannabis, 29% stimulants, 15% sedatives, 15% pornography, and 11% gambling; 11% initiated an addictive behavior or the use of an addictive substance.
Cousijn et al. (2020) [6]	- To investigate the influence of the lockdown on SUD.	Lockdown: MINI (SUD section); AUDIT; motives for cannabis use; DSM-5-CCSM; COVID-19-related worries; social contact.	- There was a significant increase in cannabis use during the first months of lockdown. However, there was no evidence of a change in substance disorder symptoms’ severity. The lockdown period was weakly associated with reductions in substance disorders symptoms.
Martinotti et al. (2020) [50]	- To evaluate the impact of the COVID-19 pandemic and the containment measures on patients with SUD and/or behavioral addictions.	Lockdown: BDI-II; DTS; IDAS; SAS; VAS (level of craving for the substance).	- A total of 23% of our sample reported moderate/severe depressive symptoms; 30% reported moderate/severe anxiety symptoms. - A positive correlation was also verified between craving and depressive symptoms, anxiety, and traumatic stress.
Yazdi et al. (2020) [51]	- To investigate addictive behavior, craving, and PTSD symptoms, as well as various COVID-19 factors in a clinical sample of patients with SUD.	Pandemic: AUDIT; PC-PTSD5; interview: physiological, economic, and substance use factors related to COVID-19.	- A total of 29% of patients were abstinent; 38% were consuming; 32% relapsed. In the total sample, psychosocial COVID-19 distress was reported by 53.5% of the patients. - A total of 8% of the participants were found to be at risk of PTSD.
*Attention-Deficit Hyperactivity Disorder*			
Becker et al. (2020) [52]	- To examine remote learning practices and difficulties during initial stay-at-home orders in adolescents with and without ADHD.	Lockdown: Home Adjustment to COVID-19 Scale; Adolescent Routines Questionnaire; COVID-19 Adolescent Symptom and Psychological Experience Questionnaire.	- The lack of routines during stay-at-home mandates, negative effects, and trouble concentrating were associated with remote learning difficulties in adolescents with ADHD.
*Schizophrenia or Other Psychotic Disorder*			
Ma et al. (2020) [53]	- To explore the influence of social isolation on the psychological characteristics of hospitalized SPD patients.	Lockdown (in patients): CPSS; HAMA; HAMD; PANSS; PSQI.	- Inpatients with schizophrenia who went through social isolation showed higher and more severe levels of stress, anxiety, and depression, and worse sleep quality afterwards than those who were not isolated.
Pinkham et al. (2020) [31]	To examine the effects of the pandemic on people with pre-existing SMI’s (including people with schizophrenia spectrum disorder or severe affective disorders).	Pandemic: EMA; MADRS; PANSS; SUMD; YMRS.	- There were no significant changes in mood or psychotic symptoms and sleep duration over time; there was a small but significant increase in the number of substances used. - Participants also referred to a significant increase in well-being post-pandemic onset.
*Tourette Syndrome or Chronic Tic Disorder*			
Mataix-Cols et al. (2020) [54]	- To investigate the impact of the COVID-19 outbreak on tic severity in people with TS or CTD.	Lockdown: self-developed Likert scale (Do you feel that your tics have worsened since the start of the coronavirus pandemic?	- Around 50% of the participants experienced a worsening in their tic symptomatology. - Increased stress and anxiety, worries about family, future, finances, changes in routines, etc., were associated with perceived tic worsening.
*Insomnia Disorder*			
Cheng et al. (2021) [55]	- To examine resilience in the sleep and stress systems during the COVID-19 pandemic in participants with ID.	Life Events Checklist; CIS; ISI; IES COVID-19; QIDS-SR; PROMIS; GMH; GPH.	- More than two-thirds of the sample (67%) reported a direct impact from the pandemic; - COVID-19-related disruptions were associated with insomnia symptoms.
*Somatic Symptom Disorder*			
De Nardi et al. (2021) [56]	- To evaluate how the COVID-19 lockdown affected adolescents with and without SSD.	Lockdown: MASC-2-SR; CDI-2-SF.	- Adolescents with SSD presented slightly lower significant anxiety raw scores and lower mean anxiety levels when compared to the controls. The physical symptoms, social anxiety, tension and restlessness scores, and levels of depression were also significantly lower in the SSD group than the controls.
*Global impact on Multiple Psychiatric Disorders*			
Favreau et al. (2021) [57]	- To evaluate the impact of the pandemic on patients with GPP who were admitted to inpatient treatment.	Pandemic: Questionnaire: changes in symptom severity, quality of life and treatment; restrictions and pandemic-related worries.	- More than 50% reported worsening of symptoms; a 40% increased need of therapeutic support; nearly 1/4 reported a setback in treatment.
Imai et al. (2021) [58]	- To investigate the impact of masks and plastic partitions on patient-doctor communication and subjective anxiety for infection in patients with psychiatric disorders.	Pandemic: questionnaire: differences in the variable’s barrier to communication and anxiety for infection for use of masks and partitions.	- A total of 91% of the patients answered that there was no change with regard to communication when the doctor was wearing a mask or using a plastic partition (83%). - Results suggest that masks and plastic partitions do not prevent patient–doctor communication, and both bring beneficial effects in reducing anxiety for infection among patients with psychiatric disorders.
Mergel et al. (2021) [59]	- To investigate social participation, inclusion and psychological well-being in adults with and without pre-pandemic mental disorders.	Pandemic and lockdown: F-INK; IMET; BSI-18; specific questions regarding their subjective concerns; subjective possibilities of events in relation to the COVID-19 pandemic.	- The group with CMD did not report an impact in terms of their social participation. The group with pre-pandemic AMD exhibited fewer impairments during the lockdown period, whereas the group without mental disorders exhibited some impairments, which were transient and decreased over time, as the limitations were lifted.
Ting et al. (2021) [60]	- To examine the prevalence of posttraumatic stress response towards COVID-19, among patients with pre-existing psychiatric illness.	Pandemic: questionnaire: impact of Effect of COVID-19 on patients’ psychological response; IES-R; PHQ-2.	- PTSD-like symptoms were reported by more than 45% of the participants. - Rumination about COVID-19 and social isolation were predictors of higher levels of PTSD-like symptoms.
Tundo et al. (2021) [61]	- To examine the psychological and psychopathological impact of the pandemic stress on patients with pre-existing MD, AD and OCD.	Pandemic and lockdown: HAMD; Y-MANIA; Y-BOCS; PAAS; BSPS.	- A total of 11% of the patients reported higher distress levels than their family and friends regarding the fear of being infected; 5% reported changes in lifestyle; and 1% also reported changes in terms of financial burden. However, 37% of the patients reported that they were better-adapted than family and friends; - Only 5% of the patients relapsed/worsened due to COVID-19 distress.
Vissink et al. (2021) [62]	- To investigate the effects of COVID-19 and the restrictive measures on stress, anxiety and loneliness in patients with psychiatric disorders.	Pandemic: BAI; BDI; PSWQ; PQL-5; PSS; WHO-ASSIST questionnaire.	- Patients reported a deterioration in general health and mental health, as well as the presence of depressive symptoms and loneliness. - A total of 26% of the affective disorder patients reported severe loneliness, versus 9% of the psychotic disorder participants.
Burrai et al. (2020) [63]	- To assess the psychological and emotional impact of isolation on patients compared to healthy controls.	Lockdown: DASS-21; BRCS; risk perception; worry about the present emergency situation; knowledge about COVID-19.	- GPP patients demonstrated lower stress compared to healthy controls; however, they presented a higher perceived risk of getting infected with COVID-19, higher levels of worry, and higher levels of anxiety relative to healthy controls.
Chang et al. (2020) [64]	- To investigate the potential factors explaining preventive COVID-19 infection behaviors among individuals with mental illness.	Pandemic: BCIS; DASS-21; FCV-19S; PCIBS; SSS-S.	- Most of the patients reported experiencing self-stigma, fear of COVID-19, trust in COVID-19 information, engagement in preventive behaviors, and psychological distress. - Less trust in the information, higher levels of self-stigma, and fear of COVID explained more psychological distress.
Hao et al. (2020) [65]	- To compare the immediate stress and psychological impact experienced by people with and without psychiatric disorder during the pandemic.	Pandemic and lockdown: IES-R; DASS-21; ISI.	- Psychiatric patients when compared to healthy controls reported a significant increase in: worries about their physical health (29% vs. 5%); moderate to severe anger and impulsivity (21% vs. 0.9%); and moderate to severe suicidal ideation (12% vs. 0.9%).
Iasevoli et al. (2020) [66]	- To examine the severity of COVID-19-related perceived stress, anxiety, depressive, and psychotic symptoms in patients with serious mental disorders, caregivers, and control group.	Pandemic and lockdown: PSS; GAD-7; PHQ-9; SPEQ.	- Patients with mental illness had higher rates of perceived stress severity, moderate to severe anxiety, and significantly higher severe depressive symptoms compared to healthy controls. - Caregivers had lower results in all evaluated components compared to healthy and mentally ill patients.
Marchitelli et al. (2020) [67]	- To identify psychological and psychosocial variables that predict weight gain in overweight patients with and without a psychiatric diagnosis.	Lockdown: BES; DASS-21; DERS; GHQ-12; PAPF-SC; risk perception.	- Among the surveyed patients affected by overweight/obesity, 50% of those without a psychiatric diagnosis and 66% of those with a psychiatric diagnosis reported weight gain during the COVID-19 lockdown. - Weight gain predictors were binge eating behaviors for patients with a psychiatric diagnosis and stress and low depression for patients without a psychiatric diagnosis.
Muruganandam et al. (2020) [68]	- To determine the impact of COVID-19 in patients with SMI and identify its relationship with their COVID-19 knowledge.	Pandemic: questionnaire: self-developed questionnaire on awareness about symptoms of COVID-19.	- A total of 73% of the SMI patients did not report any anxiety/fear of contracting COVID-19. - Impairment was noted in sleep (38%), food intake (23%), and personal care (20%). A total of 30% of the patients showed re-emergence of previous psychiatric symptoms; 14% expressed suicidal ideas during this period; and 5% reported an increase in suicidal ideas; 28% expressed feelings of physical aggression toward their caregivers; 64% reported that they were experiencing verbal and physical aggression from others; 7% reported an increase in substance use during this lockdown period.
Pan et al. (2020) [69]	- To compare the perceived mental health impact on depressive symptoms, anxiety, worry, and loneliness before and during the COVID-19 pandemic among people with/without mental disorders.	Pandemic: NESDA: QIDS; BAI; PSWQ; DJGLS; - NESDO: QIDS; BAI; DJGLS; - NOCDA: BAI; DJGLS.	- Only 3% of the participants without a history of mental health disorders scored above the threshold for moderate to severe depressive symptoms during the pandemic. By contrast, in individuals with the largest mental health disorder burden, no overall increase in symptom severity was seen.
Porcellana et al. (2020) [70]	- To evaluate the relationship between traumatic aspects of COVID-19 and clinical correlates of consecutive outpatients in a community mental health setting.	Lockdown: CGI-S; BPRS-18; IES-R; SRQ-20.	- A total of 34% of the participants reported mild symptoms of distress; 32% reported moderate symptoms of distress; and 26% reported severe symptoms of distress. SRQ-20 total score was positive in 59% of the patients, particularly in the female population aged 45–65. Actively working and SRQ-20 significantly predicted IES-R total score.
Zou et al. (2020) [71]	- To investigate the prevalence of psychiatric symptoms and pain in older, clinically stable patients with and without psychiatric disorders during the COVID-19 pandemic.	Lockdown: questionnaire: self-developed questionnaire for current fatigue; - Instruments and scales: PHQ-9; ISI; NPRS; WHOQOL-BREF.	- A total of 47% of older, clinically stable patients with psychiatric disorders reported fatigue during the COVID-19 outbreak. - A total of 69% of the fatigue group reported illness worsening during COVID-19, while 39% of the non-fatigue group reported illness worsening during COVID-19.

Note: AMD, acute mental disorder; AN, anorexia nervosa; ASI-3, Anxiety Sensitivity Index-3; AUDIT, Alcohol Use Disorder Identification Test; BAI, Beck Anxiety Inventory; BCIS, Believing COVID-19 Information Scale; BD, bipolar disorder; BDI-II, Beck Depression Inventory-II; BED, binge eating disorder; BES, Binge Eating Scale; BN, bulimia nervosa; BPRS-18, Brief Psychiatric Rating Scale; BRCS, Brief Resilient Coping Scale; BSI, Brief Symptom Inventory; BSPS, Brief Social Phobia Scale; CDI-2-SF, Children’s Depression Inventory Short Form; CGI-I, Clinical Global Impression Improvement; CGI-S, Clinical Global Impression–Severity Scale; CIES, COVID Isolation Eating Scale; CIS, Coronavirus Impact Scale; C19P-S, COVID-19 Phobia Scale; CMD, chronic mental disorder; CPSS, Chinese Perceived Stress Scale; CRIES-13, Children’s Impact of Event Scale; CSS, COVID Stress Scales; CTQ-SF, Childhood Trauma Questionnaire—Short Form; CY-BOCS, Children’s Yale–Brown Obsessive Compulsive Scale; DASS-21, Depression, Anxiety and Stress Scale-21; DD, depressive disorder; DERS, Difficulties in Emotional Regulation Scale; DJGLS, De Jong Gierveld Loneliness Scale; DSM-5-CCSM, DSM-5 self-rated level 1 cross-cutting symptom measure—adult; DTS, Davidson Trauma Scale; ECR-R, Experiences in Close Relationships—Revised; EDE-Q, Eating Disorder Examination Questionnaire; EDI-2, Eating Disorders Inventory-2; EMA, Ecological Momentary Assessments; FCV-19s, Fear of COVID-19 scale; F-INK, Measure of Participation and Social Inclusion for Use in People with a Chronic Mental Disorder; GAD-7, Generalized Anxiety Disorder Scale; GAF, Global Assessment of Function; GMH, Global Mental Health; GPH, Global Physical Health; GHQ-12, General Health Questionnaire-12; HDRS, Hamilton Depression Rating Scale; HAMA, Hamilton Anxiety Scale; HAMD, Hamilton Depression Scale; HAM-D, Hamilton Depression Rating Scale; HCQ-54, Health Concerns Questionnaire-54; IDAS, Irritability Depression Anxiety Scale; IES COVID-19, Impact of Events Scale; IES-R, Impact of Event Scale-Revised; IMET, Index for the Assessment of Health Impairments; ISI, Insomnia Severity Index; K10, Kessler 10 Psychological Distress Scale; LEC, Life Event Checklist; MADRS, Montgomery–Asberg Depression Rating Scale; MASC-2-SR, Multidimensional Anxiety Scale for Children Self Report; MDD, major depressive disorder; NESDA, Netherlands Study of Depression and Anxiety; NESDO, Netherlands Study of Depression in Older Persons; NOCDA, Netherlands Obsessive Compulsive Disorder Association Study; NPRS, Numeric Pain Rating Scale; OCI-R, Obsessive Compulsive Inventory—Revised; OCS, Obsession with COVID-19 scale; PAAS, Panic Attack and Anticipatory Anxiety Scale; PANSS, Positive and Negative Syndrome Scale; PAPF-SC, Parents’ Assessment of Protective Factors: Social Connections Subscale; PCIBS, Preventive COVID-19 Infection Behaviors Scale; PC-PTSD5, Primary Care PTSD Screen for DSM-5; PHQ, Patient Health Questionnaire; PROMIS, Patient-Reported Outcomes Measurement Information System; PCL-5, Post-Traumatic Stress Disorder derived from DSM-V; PSQI, Pittsburg Sleep Quality Index; PSS, Perceived Stress Scale; PSWQ, Penn State Worry Questionnaire; PTSD, post-traumatic stress disorder; PTSS, post-traumatic stress symptoms; QIDS-SR, Quick Inventory of Depressive Symptomatology; SAS, Self-rating Anxiety State; SCL-90-R, Symptom Checklist-90-Revised; SHAI, Short Health Anxiety Inventory; SMI, severe mental illness; SPEQ, Specific Psychotic Experience Questionnaire; SRQ-20, Self-Report Questionnaire; SSS-S, Self-Stigma Scale-Short; STAI, State-Trait Anxiety Inventory; SUMD, Scale to Assess Unawareness of Mental Disorder; TCI-R, Temperament and Character Inventory-Revised; VAS, Visual Analogue Scale; VOCI, Vancouver Obsessional Compulsive Inventory; XS, Xenophobia Scale; WHOQOL-BREF, World Health Organization Quality of Life-brief version; WMD, without a mental disorder; YMRS, Young Mania Rating Scale; Y-BOCS, Yale–Brown Obsessive Compulsive Scale; YFAS-2, Yale Food Addiction Scale 2.0; YMRS, Young Mania Rating Scale.

**Table 4:** Summary of main findings.

Psychiatric Disorders	Main Results	Major Concerns or Potential Risk Factors
Mood	No or mild/moderate impact. BD: Greater impact when compared to healthy controls; Slower recovery over time. People with MDD were more likely to develop PTSD-like symptoms, as compared to healthy controls. Reduced negative impact when comorbid psychotic symptoms were present.	Frustration due to restrictions was the most common stressor. Work and financial difficulties. Fear of infection.
Anxiety	Modest impact, although greater for those with comorbid conditions. Some patients reported improvement as compared to evaluation prior to the beginning of the pandemic restrictions. Individuals with GAD and PD reported higher levels of stress associated with COVID-19 pandemic.	Anxiety sensitivity may be a potential vulnerability factor for COVID-19-related mental health impacts. Reduced social functioning and quality of life were positively correlated to the greatest impact of the COVID-19 restrictions.
Obsessive compulsive	Significant worsening of OCD symptoms. Emergence of COVID-related OC symptoms in some patients.	Higher risk of OCD symptoms worsening: Presence of pre-pandemic depression; Higher Y-BOCS scores; contamination/washing symptoms; Lower perceived social support.
Eating	Significant ED worsening symptoms: Some studies showed that the effect was higher in people with AN; Other studies showing that it was in people with BN. Significant negative interference in the recovery process. Significant increase in binge eating episodes.	ED worsening symptoms related to: Dysfunctional coping strategies; Lower self-directedness; Personality trait; History of adverse childhood experiences. Fear for the safety of loved ones, as well as worsening of depressive and anxiety symptoms might lead to an increase in binge eating episodes.
Substance use and addiction	Mixed results in the literature of worsening or improvement of substance use or addictive behaviors. The impact of the lockdown on mental health in cannabis users might have been minimal.	Greater impact of the pandemic: Trauma exposure; Perceived stress and isolation; Income reduction and financial uncertainty; Alcohol abuse. Isolation, anxiety, depression, and living alone may be associated with a higher risk of relapsing.
Attention-deficit hyperactivity	Adolescents with ADHD experienced greater negative effects and more difficulties concentrating.	Remote learning seems to be more challenging for adolescents with ADHD, as compared to healthy individuals.
Schizophrenia Spectrum	Greater psychological (depression, anxiety, and sleep quality) impact for those that were socially isolated during hospitalization due to pandemic restrictions	Social isolation associated with burden.
Tourette Syndrome or Chronic Tic Disorder	Tic worsening.	Increased stress and anxiety: Preoccupations about finances, future, and family; Confinement and lack of physical exercise/activity; Fewer distractions, change in or lack of routines, having to go to work, and being exposed to the public. Possible stigma related to: Coughing tics; Exacerbation of the tics while using a mask; Increase in self-injury behaviors.
Insomnia	Moderate to severe insomnia.	
Somatic Symptom Disorders	Adolescents with SSD experienced fewer physical, depressive, and anxiety symptoms, as compared to healthy controls, during the COVID-19 blockade period.	Lockdown may be related to reduced symptomatology in adolescents with SSD due to reduced social distress.

## Data Availability

Not applicable.

## Supplementary Materials

The following supporting information can be downloaded at: https://www.mdpi.com/article/10.3390/ijerph19095140/s1. File S1: Search Strategy—mesh terms.
