# Supplementary material for: The Acute Impact of the Early Stages of COVID-19 Pandemic in People with Pre-Existing Psychiatric Disorders: A Systematic Review
Source: Int J Environ Res Public Health. 2022 Apr 23;19(9):5140. doi: 10.3390/ijerph19095140 (PMC9104538; doi:10.3390/ijerph19095140)
Supplement: Supplementary file 1 [file ijerph-19-05140-s001.zip › ijerph-1545461-supplementary.pdf]

Supplementary material

Search Strategy - mesh terms:

The mesh terms used to psychiatric disorders were: Anxiety OR Phobic disorders OR Panic disorder OR Agoraphobia OR Anxiety disorders OR Mood disorders OR Depressive disorder OR Depression OR Major depressive disorder OR Major depressive episode OR Cyclothymic disorder OR Bipolar disorder OR Trauma and Stressor Related Disorders OR Adjustment Disorders OR Post-Traumatic Stress Disorder OR Stress Disorders, Traumatic OR Stress Disorders, Traumatic, Acute OR Gambling OR Gambling disorder OR Drug addiction OR Addiction OR Crack Cocaine OR Crack-cocaine OR Alcohol Abuse OR Cocaine Abuse OR Marijuana Abuse OR Cocaine-Related Disorders OR Amphetamine-Related Disorders OR Alcohol-Related Disorders OR Substance-Related Disorders OR Feeding and Eating Disorders OR Eating Disorders OR Anorexia Nervosa OR Binge-Eating Disorder OR Bulimia Nervosa OR Food Addiction OR Avoidant Restrictive Food Intake Disorder OR Night Eating Syndrome OR Pica OR Neurodevelopmental Disorders OR Attention Deficit and Disruptive Behavior Disorders" OR "Attention Deficit Disorder with Hyperactivity" OR "Attention-deficit hyperactivity disorder" OR "Conduct Disorder" OR "Autism Spectrum Disorder" OR "Asperger Syndrome" OR "Autistic Disorder" OR "Tic Disorders" OR "Tourette Syndrome" OR "Developmental Disorders" OR "Personality Disorders" OR "Antisocial Personality Disorder" OR "Borderline Personality Disorder" OR "Compulsive Personality Disorder" OR "Dependent Personality Disorder" OR "Histrionic Personality Disorder" OR "Paranoid Personality Disorder" OR "Passive-Aggressive Personality Disorder" OR "Schizoid Personality Disorder" OR "Schizotypal Personality Disorder" OR "Schizophrenia Spectrum and Other Psychotic Disorders" OR Schizophrenia OR "Psychotic Disorders" OR "Paranoid Disorders OR "Affective Disorders, Psychotic" OR "Mental disorders" OR "Disruptive, Impulse Control, and Conduct Disorders" OR "Anxiety, Separation" OR "Sexual Dysfunctions, Psychological" OR "Sleep Wake Disorders" OR "Somatoform Disorders" OR "Neurocognitive Disorders" OR "Dementia" OR "Alzheimer Disease" OR "Obsessive-Compulsive Disorder" OR "Obsessive-Compulsive Spectrum Disorders". Afterwards, this search was merged using "AND" with the main keywords related to the COVID-19 pandemic: "COVID-19" OR "SARS-CoV-2" OR "pandemics" OR "coronavirus".
